# Unveiling the tendon toxicity spectrum of anastrozole, letrozole, and exemestane: a real-world pharmacovigilance study

**DOI:** 10.3389/fonc.2026.1825180

**Published:** 2026-07-07

**Authors:** Shuai Zhao, Lu-Yao Xu, Ping Chen, Su Zhang, Kai-Li Mao

**Affiliations:** 1 Department of Emergency Medicine, Nanyang Central Hospital, Nanyang, China; 2 Department of Pharmacy, The Quzhou Affiliated Hospital of Wenzhou Medical University, Quzhou People’s Hospital, Quzhou, China; 3 Department of Pharmacy, Center for Clinical Pharmacy, Cancer Center, Zhejiang Provincial People’s Hospital, Affiliated People’s Hospital, Hangzhou Medical College, Hangzhou, China

**Keywords:** data mining, FAERS, pharmacovigilance, tendon disorders, third generation aromatase inhibitors

## Abstract

**Background:**

Aromatase inhibitors have been successfully employed in clinical practice for the treatment of breast cancer (BC); however, the accompanying tendon disorders has often been underestimated. This study aimed to conduct a comprehensive empirical investigation and assess tendon disorders associated with third-generation aromatase inhibitors using data from the FDA’s FAERS database. The goal was to provide a theoretical foundation for the rational application of these drugs in clinical settings.

**Methods:**

Reports of tendon disorders related to third-generation aromatase inhibitors were retrieved from the FAERS database, covering the period from the first quarter of 2004 to the first quarter of 2024. The data were further analyzed using proportional analysis and a Bayesian approach to detect signals of tendon disorders induced by the three drugs. Additionally, the clinical characteristics, onset time, correlation, and stratification analysis of tendon disorders associated with the three drugs were examined.

**Results:**

Positive signals for tendon disorders associated with third-generation aromatase inhibitors were identified. At the preferred term (PT) level, twelve positive signals were detected, with trigger finger and tenosynovitis stenosans presenting a significant signal. In terms of onset time, letrozole exhibited the earliest onset at 74 days, whereas exemestane showed the latest onset at 243.5 days. After excluding the effect of combination drugs, a clear association between third-generation aromatase inhibitors and tendon disorders was observed.

**Conclusion:**

Analysis of the FAERS database has identified risk trends for tendon disorders associated with third-generation aromatase inhibitors. Furthermore, the study reveals varying degrees of tendon toxicity related to these inhibitors, including distinct time-to-onset profiles and drug-specific signal intensities, providing valuable insights into associated tendon disorders in clinical practice.

## 1 Introduction

As per the 2020 global data report, the incidence of newly diagnosed cases of breast cancer (BC) surpassed that of lung cancer for the first time. With 11.7% of all cancer cases, BC emerged as the most common malignancy worldwide (1). BC, mostly dependent on estrogen or progesterone, is a deadly form of malignancy especially in the case of postmenopausal females (2, 3). Sequential endocrine therapy is commonly used to treat over 70% of patients with BC who have illness that is both hormone receptor-positive and human epidermal growth factor receptor 2-negative or positive (4). Endocrine therapy has significantly improved outcomes for patients with early- and advanced-stage hormone-receptor (HR)-positive BC. Two main approaches are currently used: modulation of estrogen receptor (ER) by selective estrogen receptor modulators (SERMs) such as tamoxifen, and inhibition of aromatase enzyme by aromatase inhibitors (AIs) (5–7). The results of several large clinical trials compared tamoxifen with third-generation AIs and established AI treatment in postmenopausal women with breast cancer (8). AIs are more effective than SERMs because they block both the genomic and nongenomic activities of ER.

Aromatase is the key enzyme which converts the enone ring of androgen precursors, such as testosterone, into a phenol for the synthesis of estrogen. AIs are used to either block the production of estrogen or block the action of estrogen on receptors (9). In the case of premenopausal women, most of the estrogen is produced in the ovaries but in postmenopausal women, it is mainly produced in peripheral tissues of the body (10). It has been found that HR-positive BC responds to estrogen production at the site of cancer, like adipose tissue of the breast, which can be inhibited by AIs (11). Estrogen deprivation is a very effective treatment for patients with HR-positive BC. By blocking or deactivating aromatase, aromatase inhibitors (AIs) considerably reduce plasma estrogen levels in postmenopausal women, offering a novel method of endocrine therapy for BC (12).

Currently, postmenopausal BC patients receive conventional treatment with third-generation AIs. For cancer patients with postmenopausal estrogen receptor-positive cancer, which makes up the majority of BC patients, these drugs successfully challenge tamoxifen, which was once the recommended course of treatment (13). A new generation of aromatase inhibitors, including non-steroidal, reversible inhibitors such as Anastrozole and Letrozole, as well as a steroidal, irreversible inhibitor like Exemestane, was developed in the mid-1990s (14). Compared to tamoxifen, third-generation AIs are associated with a lower incidence of potentially fatal adverse events (AEs), such as thromboembolic episodes and the emergence of endometrial cancer. However, AIs exhibit a distinctive musculoskeletal safety profile, which has received increasing attention. Musculoskeletal symptoms induced by AIs include arthralgia, joint stiffness, bone pain, tenosynovitis, tendinopathy, and an increased risk of osteoporosis (15–19). Among these, tendon disorders such as trigger finger and stenosing tenosynovitis remain underrecognized in clinical practice despite their potential to cause persistent functional impairment. The underlying mechanisms of AI-associated musculoskeletal and tendon disorders are not fully elucidated but are likely multifactorial. Estrogen plays a protective role in maintaining tendon structure and function by promoting collagen synthesis and reducing inflammatory responses. Estrogen receptors have been identified in the retinaculum, annular ligaments, and tenocytes (20). Estrogen deprivation induced by AIs may lead to alterations in these tissues, contributing to tenosynovitis and tendinopathy. Compared with tamoxifen, AIs are associated with significantly higher risks of osteoporosis, osteopenia, fracture, and arthralgia/myalgia; these musculoskeletal adverse events are mainly caused by AI-induced systemic and local estrogen deficiency, which accelerates bone turnover, promotes osteoclastogenesis, impairs chondroprotection, increases joint effusion and tenosynovial changes, and enhances pain perception, ultimately leading to clinical bone loss and arthralgia syndrome (19). The U.S. Food and Drug Administration (FDA) has revised its drug safety information for AIs several times, warning that such drugs can cause decreased bone density and neurological deterioration, as well as revising the drug inserts. Recently, the European Medicines Agency (EMA) has requested that a risk warning for tendonitis and tendon rupture be added to the European product description for letrozole, but has not ruled out the association of other third-generation AIs with the risk of tendon disorders. Additionally, the website of the Canada’s department of Health posted the information to warn the risk of tendon disease with the third generation AIs in January 2023 (21). According to these researches, third-generation AIs may cause tendon disorders, which can cause lasting harm. Unfortunately, there are currently insufficient case reports and pre-market clinical investigations about third generation AIs and tendon disorders, making it impossible to draw firm conclusions about the relationship between third generation AIs and tendon disorders in practical settings.

The potential adverse effects of the drug should be taken into account while choosing a specific treatment plan for BC patients, in order to obtain a very long survival. It is vital to investigate the connection between different third generation AIs and tendon disorders given the widespread clinical use. An extensive amount of real-world data is gathered by the FDA Adverse Event Reporting System (FAERS), a spontaneous reporting system that gathers AE reports worldwide (22). It has been widely used to identify AE risk signals. This study aimed to evaluate the risk of tendon disorders related with different third generation AIs using standardized data from the FAERS.

## 2 Data and methods

### 2.1 Data sources

The FDA’s post-marketing surveillance program for pharmaceuticals and therapeutic biological products is supported by the voluntary reports that are incorporated into the FAERS database from a variety of sources, including patients, pharmacists, healthcare professionals, and pharmaceutical corporations. The FAERS database was launched by the U.S. FDA in 2004 for post-marketing safety surveillance. To ensure the inclusion of the latest reports, we extracted all FAERS reports recorded between the first quarter of 2004 and the first quarter of 2024. It includes a variety of data, such as patient demographics, medication usage, adverse reaction information, reporting sources, length of therapy, drug indications, and patient outcomes. Seven different types of data documents were found in the FAERS data files: DRUG (drug information), OUTC (patient outcomes), RPSR (report sources), THER (therapy start and end dates for reported drugs), INDI (indications for drug administration), and DEMO (demographic and administrative information). In the FAERS database architecture, these files were linked together by unique identifying numbers like PRIMARYID.

### 2.2 Data extraction

In this study, we selected the following drugs for research: anastrozole, letrozole, and exemestane. All the AEs were classified using the Medical Dictionary of Regulatory Activities (MedDRA; version 25.1), and the PTs were allocated according to systemic organ classes (SOCs). MedDRA has five levels from low to high: the lowest-level term (LLT), preferred term (PT), high-level term (HLT), high-level group term (HLGT), and system organ class (SOC). The PTs of all the tendon adverse events were acquired, with the SOC as “tendon disorders”.

**Figure 1:**
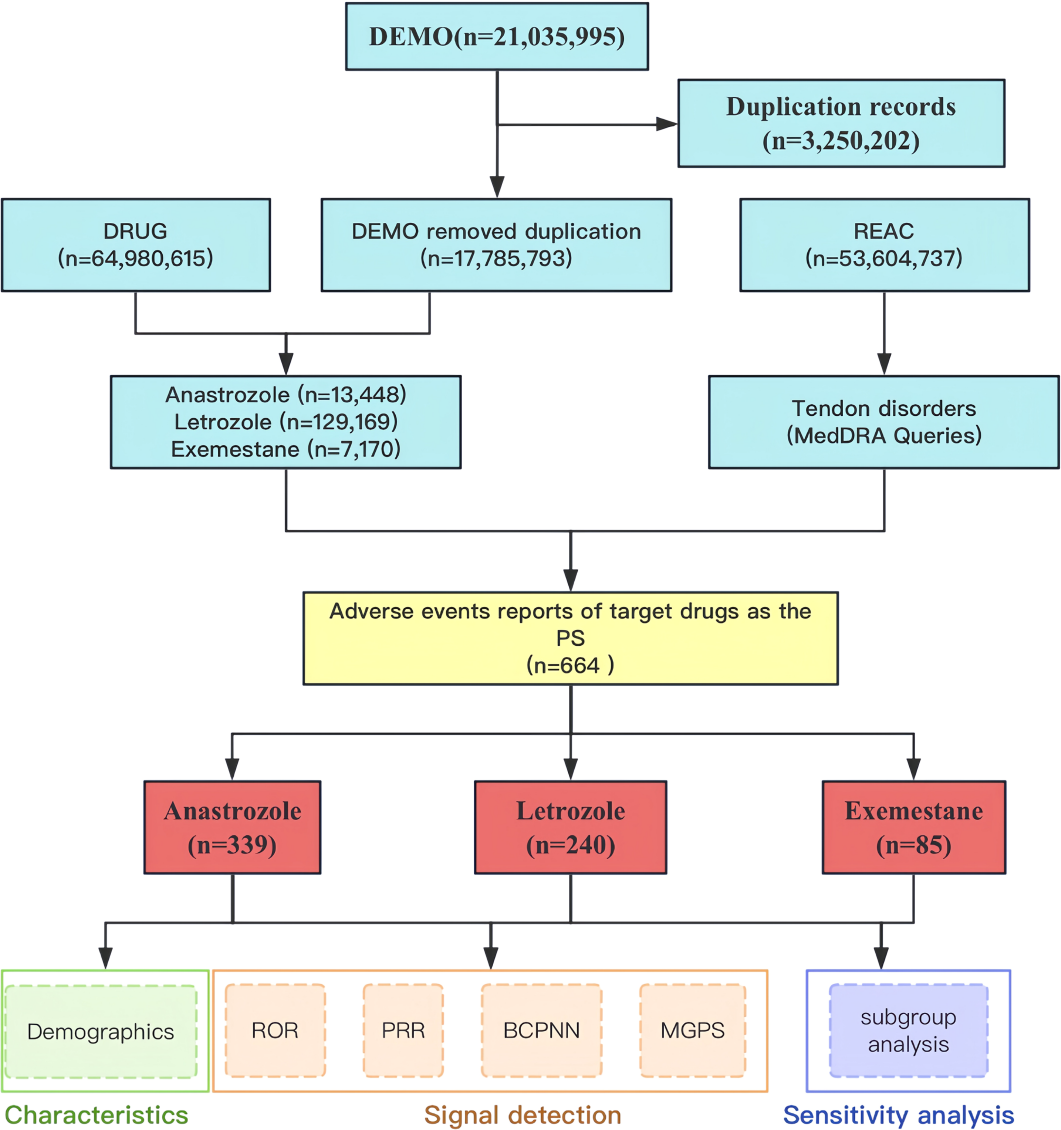
The flow diagram of third generation AIs-related tendon disorders from FAERS database. AIs, aromatase inhibitors; BCPNN, Bayesian confidence propagation neural network; DEMO, demographic and administrative information; MGPS, multiple Gamma Poisson reduction method; PRR, proportional reported odds ratio; PS, principal suspect; REAC, adverse reaction; ROR, reported odds ratio.

The comprehensive screening process is illustrated in **Figure 1**. If two reports had the same adverse event, individual safety report(ISR) number, date of delivery, medicine, indication, sex, reporting country, and age, they were deemed duplicates. The remaining reports were further screened by designating the principal suspect (PS) as the main selection criterion (Third generation AIs), after probable tendon disorders that could result from concurrent drugs and drug interactions were excluded. Following the aforementioned deduplication procedure, additional analysis was conducted using the remaining reports.

### 2.3 Data analysis

In this study, we employed proportional disequilibrium method and Bayesian method, which are widely used for signal detection in pharmacovigilance. The proportional disequilibrium method involves a comparison of the occurrence proportions of adverse events between a target drug and all other drugs. Both the proportional reported odds ratio (PRR) and the reported odds ratio (ROR) are included in this analysis (23). Additionally, the Bayesian method incorporates two prominent algorithms: the Bayesian confidence propagation neural network (BCPNN) and the multiple Gamma Poisson reduction method (MGPS) (24). To enhance the credibility of the correlation analysis between drugs and adverse events, this study employed four distinct algorithms: ROR, PRR, BCPNN, and MGPS. These algorithms were employed to quantify the correlation between third generation AIs and tendon disorders, respectively. Drawing from the four-cell table of the ratio imbalance method (**Supplementary Table 1**), as well as the Bayesian method, the formulas and signal detection criteria for these four algorithms were delineated in **Table 1** (25). In general, a higher algorithmic value signifies a more pronounced signal, indicating a more robust correlation between the drug and the occurrence of adverse events.

**Table 1:** Formulas and thresholds of the four algorithms.

Algorithms	Equation	Criteria
ROR	$\text{ROR}=\frac{a/c}{b/d}$ $\text{SE}\left(1\text{nROR}\right)=\sqrt{\frac{1}{a}+\frac{1}{b}+\frac{1}{c}+\frac{1}{d}}$ $95\%\text{CI}=e^{1\text{n}\left(\text{ROR}\right)\pm 1.96\text{se}}$	a ≥ 3, 95%CI (low limit)> 1
PRR	$\text{PRR}=\frac{a/\left(a+b\right)}{c/\left(c+d\right)}$ $\text{SE}\left(1\text{nPRR}\right)=\sqrt{\frac{1}{a}-\frac{1}{a+b}+\frac{1}{c}-\frac{1}{c+d}}$ $95\%\text{CI}=e^{1\text{n}\left(\text{PRR}\right)\pm 1.96\text{se}}$	a ≥ 3, 95%CI (low limit)> 1
BCPNN	$\begin{array}{l} \text{IC = }\log_{2}\;\frac{a\;\left(a+b+c+d\right)}{\left(a+b\right)\;\left(a+c\right)}\\ \text{E }\left(\text{IC}\right)\;=\;\log_{2}\;\frac{\left(a+\gamma 11\right)\;\left(a+b+c+d+a\right)\;\left(a+b+c+d+\beta \right)}{\left(a+b+c+d+\gamma \right)\;\left(a+b+a1\right)\;\left(a+c+\beta 1\right)}\\ \text{V }\left(\text{IC}\right)\;=\;\frac{1}{{\left(\ln2\right)}^{2}}\;[\;\frac{\left(a+b+c+d\right)\;-a+\gamma -\gamma 11}{\left(a+\gamma 11\right)\;\left(1+a+b+c+d+\gamma \right)}\;+\\ \;\frac{\left(a+b+c+d\right)-\;\left(a+b\right)+a-\alpha 1}{\left(a+b+\alpha 1\right)\;\left(1+a+b+c+d+\alpha \right)}+\\ \;\frac{\left(a+b+c+d+\alpha \right)-\;\left(a+c\right)+\beta -\beta 1}{\left(a+b+\beta 1\right)\;\left(1+a+b+c+d+\beta \right)}]\\ \gamma =\gamma 11\frac{\left(a+b+c+d+\alpha \right)\;\left(a+b+c+d+\beta \right)}{\left(a+b+\alpha 1\right)\;\left(a+c+\beta 1\right)}\\ \text{IC}-2SD=E\left(\text{IC}\right)\;-\;\sqrt{\text{V }\left(\text{IC}\right)} \end{array}$	IC025 > 0
MGPS	$\text{EBGM}=\frac{a\left(a+b+c+d\right)}{\left(a+c\right)\left(a+b\right)}$ $\text{SE}\left(1nEBGM\right)=\sqrt{\frac{1}{a}+\frac{1}{b}+\frac{1}{c}+\frac{1}{d}}$ $95\%\text{CI}=e^{1n\left(\text{EBGM}\right)\pm 1.96\text{se}}$	EBGM05 > 2

Equation: a, number of reports containing both the target drug and target adverse drug reaction; b, number of reports containing other adverse drug reaction of the target drug; c, number of reports containing the target adverse drug reaction of other drugs; d, number of reports containing other drugs and other adverse drug reactions. 95%CI, 95% confidence interval; N, the number of reports; X2, chi-squared; IC, information component; IC025, the lower limit of 95% CI of the IC; E(IC), the IC expectations; V(IC), the variance of IC; BCPNN, Bayesian Confidence Propagation Neural Network; EBGM, empirical Bayesian geometric mean; MGPS, multi-item gamma Poisson shrinker; EBGM05, the lower limit of 95% CI of EBGM; PRR, Proportional Reporting Ratio.

Time to onset was defined as the duration between the initiation of third generation AIs treatment and the occurrence of tendon disorders. The time of adverse event occurrence was computed using the following formula: (Time of occurrence = Time adverse event date - Initiation date of the use of third generation AIs. Instances of date entry errors (where EVENT_DT preceded START_DT) or inaccuracies were excluded (22, 26). To illustrate the time to onset of tendon disorders, we presented the median number of days and the corresponding interquartile range (IQR) encompassing the first quartile to the third quartile. We analyzed the signals of tendon disorders related to third generation AIs.

### 2.4 Statistical analysis

Information regarding patient demographics, including gender, age, reporting region, and outcomes, were documented in tendon disorders reports with the use of third generation AIs. Combining third generation AIs with other medications is a frequent approach to obtain increased therapeutic efficacy in the treatment regimen of hormone receptor-positive BC. Furthermore, the use of numerous drugs is not uncommon among patients due to the possibility of multiple co-occurring medical disorders. The development and course of tendon disorders may be affected by certain co-administered drugs. To reduce these possible biases and improve the reliability of our results, we analyzed the top 10 concomitant medications and stratified by combination therapy and without combination therapy. Ovarian suppression therapy was not included among the top 10 concomitant medications and was not further analyzed due to its rare use in the postmenopausal population receiving AIs. R software (version 4.1.2) and Microsoft Excel 2019 were used for all statistical analysis and data mining.

## 3 Results

### 3.1 Clinical characteristics

We obtained 664 cases of tendon disorders with the “primary suspect drug” of the third generation AIs from the FAERS database. The clinical characteristics of the patients are summarized in **Table 2**. The majority of patients were females, the proportion of females with anastrozole, exemestane and letrozole was 94.1% (319/339), 94.1% (80/85), and 96.3% (231/240) respectively. In terms of age, the most frequently affected group was 45–64 years, representing 43.0% (146/339) of anastrozole reports, 43.4% (37/85) of exemestane reports, and 39.2% (94/240) of letrozole reports. Regarding the country of origin, the United States (58.4% of reports for anastrozole and 55.3% of reports for exemestane) and the United Kingdom (25.0% of reports for letrozole) constituted the largest proportion of reports across all three drugs. Regarding outcomes of AEs, the primary outcome for anastrozole (33.6%), exemestane (36.5%), and letrozole (56.7%) was “other serious outcomes”, succeeded by disability and hospitalization. It is worth noting that the serious outcomes include death, life-threatening, hospitalization, disability, and other serious outcomes.

**Table 2:** Clinical characteristics of third generation AIs-related tendon disorders.

Characteristic	Anastrozole, N = 339	Exemestane, N = 85	Letrozole, N = 240
Weight/kg, Median(IQR)	71 (65, 84)	71 (62, 88)	77 (63, 83)
Unknown	161	32	154
Sex, n (%)			
F	319 (94.1%)	80 (94.1%)	231 (96.3%)
M	3 (0.9%)	0 (0.0%)	2 (0.8%)
Unknown	17(5.0%)	5(5.9%)	7(2.9%)
Occupation of reporters, n (%)			
Consumer	149 (44.0%)	41 (48.2%)	113 (47.1%)
Pharmacist	26 (7.7%)	8 (9.4%)	27 (11.3%)
Lawyer	1 (0.3%)	1 (1.2%)	0 (0.0%)
Physician	51 (15.0%)	18 (21.2%)	46 (20.0%)
Other health-professional	36 (10.6%)	10 (11.8%)	44 (19.2%)
Unknown	76(22.4%)	7(8.2%)	10(4.2%)
Reported countries (Top 3), n (%)			
1	US 198 (58.4% )	US 47 (55.3%)	GB 60 (25.0%)
2	GB 72 (21.2% )	DE 10 (11.8%)	US 45 (18.8%)
3	CA 9 (2.7%)	FR 7 (8.2%)	DE 30 (12.5%)
Outcomes, n (%)			
Death	0 (0.0%)	0 (0.0%)	1 (0.4%)
Disability	95 (28.0%)	16 (18.8%)	42 (17.5%)
Hospitalization	19 (5.6%)	11 (12.9%)	30 (12.5%)
Life-Threatening	1 (0.3%)	2 (2.4%)	0 (0.0%)
Required Intervention	3 (0.9%)	1 (1.2%)	0 (0.0%)
Other Serious	114 (33.6%)	31 (36.5%)	136 (56.7%)
Unknown	107(31.6%)	24(28.2%)	31(12.9%)
Age_group/ years, n (%)			
<18	0 (0.0%)	0 (0.0%)	3 (1.3%)
18-44	9 (2.7%)	1 (1.2%)	7 (2.9%)
45-64	146 (43.0%)	37 (43.4%)	94 (39.2%)
65-84	89 (26.3%)	23 (27.1%)	50 (20.8%)
≥85	0 (0.0%)	1 (1.2%)	2 (0.8%)
Unknown	95(28.0%)	23(27.1%)	84(35.0%)

AIs, aromatase inhibitors; US, United States; GB, United Kingdom; CA, Canada; DE, Germany; FR, France; IQR, interquartile range.

### 3.2 Annual distribution of tendon disorders reports related to third generation AIs

**Figure 2:**
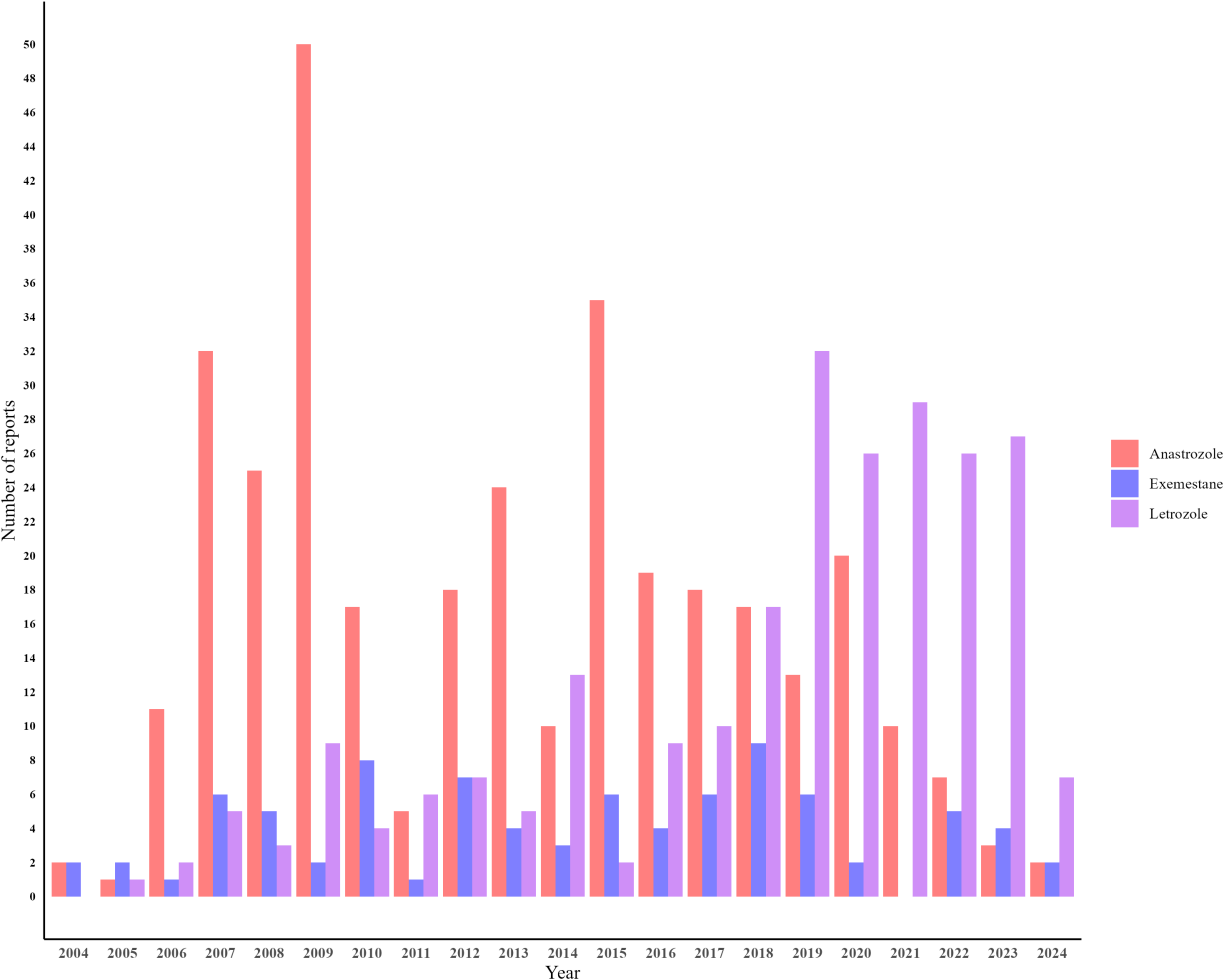
The number of reported cases of third generation AIs-related tendon disorders per year. 2024 refers to the data for the first quarter of 2024. AIs, aromatase inhibitors.

A total of 21,035,995 AE reports were involved in the FAERS database from the first quarter of 2004 to the first quarter of 2024. After excluding duplicate reports, 664 cases of third generation AIs-related tendon disorders were identified. Among these, there were 339 recorded cases of anastrozole-related tendon disorders, 85 recorded cases of exemestane-related tendon disorders and 240 recorded cases of letrozole-related tendon disorders (**Figure 2**). Exemestane presents minimal number of reported cases, ranging from 0 to 9 cases, with little fluctuation in the number of reported cases from year to year. This discrepancy is more likely attributable to lower real-world prescription frequency and underreporting rather than a genuine absence of tendon toxicity, as exemestane still generated a positive disproportionality signal (**Table 3**). In contrast, anastrozole has the highest number of reported cases, ranging from 1 to 50 cases, and fluctuates widely. In recent years, there has been a significant increase in the number of reported cases of letrozole-related tendon disorders compared to the previous years before 2017. Overall, the number of tendon diseases linked to these third-generation aromatase inhibitors that have been described the importance of monitoring these conditions.

**Table 3:** Signal detection of third generation AIs-related tendon disorders.

Drugs	Number of reports	ROR (95% Cl)	PRR (χ2)	EBGM (EBGM05)	IC (IC025)
Anastrozole	339	7.64 ( 6.9 - 8.46 )	7.59( 2144.42 )	7.54 ( 6.93 )	2.92( 1.25 )
Exemestane	85	4.4( 3.6 - 5.38 )	4.39 ( 248.05 )	4.38 ( 3.7 )	2.13 ( 0.46 )
Letrozole	240	3.69( 3.28 -4.16 )	3.68( 527.35 )	3.67 ( 3.32 )	1.88( 0.21 )

AIs, aromatase inhibitors.

### 3.3 Tendon disorders signal mining results of third generation AIs

The number of tendon disorders associated with each specific third generation AIs, as well as the corresponding ROR, PRR, EBGM05 and IC025 are shown in **Table 3**. In this study, four algorithms, namely ROR, PRR, BCPNN, and MGPS, were employed to analyze the correlation between anastrozole, exemestane or letrozole-related tendon disorders. According to their respective signal detection criteria, all four algorithms consistently indicated positive signals for anastrozole, exemestane and letrozole in relation to the tendon disorders. Compared to other AIs, anastrozole (N = 339, ROR = 7.64, 95% CI [6.9–8.46], PRR = 7.59, X2 = 2144.42) showed the highest safety concerns regarding the tendon disorders, exemestane (N = 85, ROR = 4.4, 95% CI [3.6–5.38], PRR = 4.39, X2 = 248.05) showed higher safety concerns regarding the tendon disorders, while letrozole (N = 240, ROR = 3.69, 95% CI [3.28–4.16], PRR = 3.68, X2 = 527.35) showed the lowest safety concerns regarding the tendon disorders.

**Figure 3:**
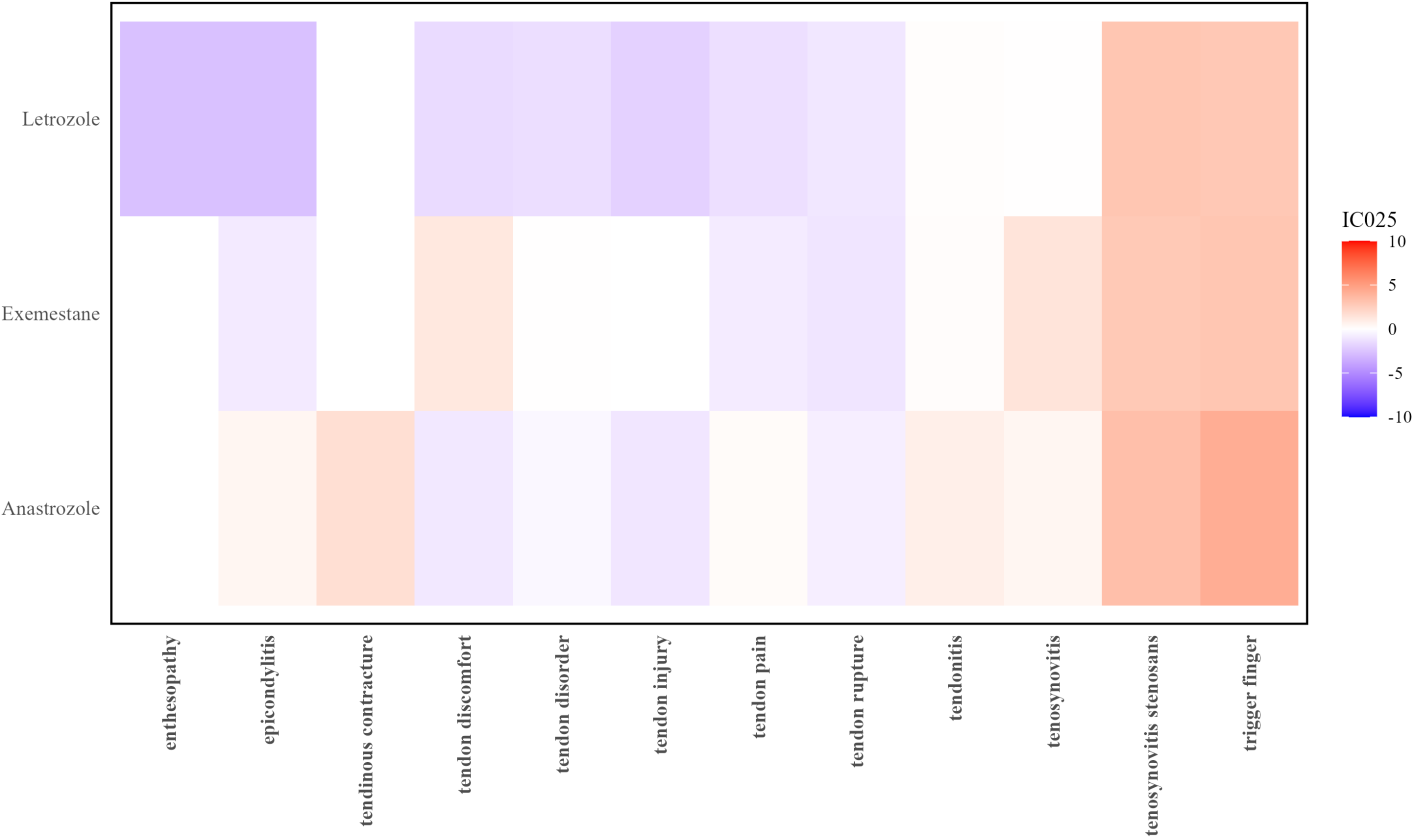
Heatmap showing the associations between third generation AIs and tendon disorders. AIs, aromatase inhibitors.

In order to better scan the correlation for third generation AIs and tendon disorders, we explored the spectrum of tendon disorders at the PT level. A total of 12 positive signals at the PT level were identified (**Figure 3**). Anastrozole and exemestane were involved in more PT signals, with 7 (IC_025_ range: 0.21–4.23) and 6 signals (IC_025_ range: 0.05–3), respectively. Anastrozole presented a significant correlation with trigger finger (IC_025_: 4.23), tenosynovitis stenosans (IC_025_: 3.31) and tendinous contracture(IC_025_: 1.69). For exemestane, trigger finger (IC025: 3.0), tenosynovitis stenosans (IC025: 2.83), tenosynovitis(IC025: 1.41) and tendon discomfort(IC025: 1.23) had strong positive signals. Despite only 4 positive signals for letrozole, tenosynovitis stenosans (IC025: 2.99) and trigger finger (IC025: 2.88) were significant signals. It is worth mentioning that trigger finger and tenosynovitis stenosans were observed as a remarkable association with all three drugs. Several specific signals were unveiled, including tendinous contracture linked to anastrozole (IC025: 1.69) and tendon discomfort associated with exemestane(IC025: 1.23).

### 3.4 Time to onset of tendon disorders

**Figure 4:**
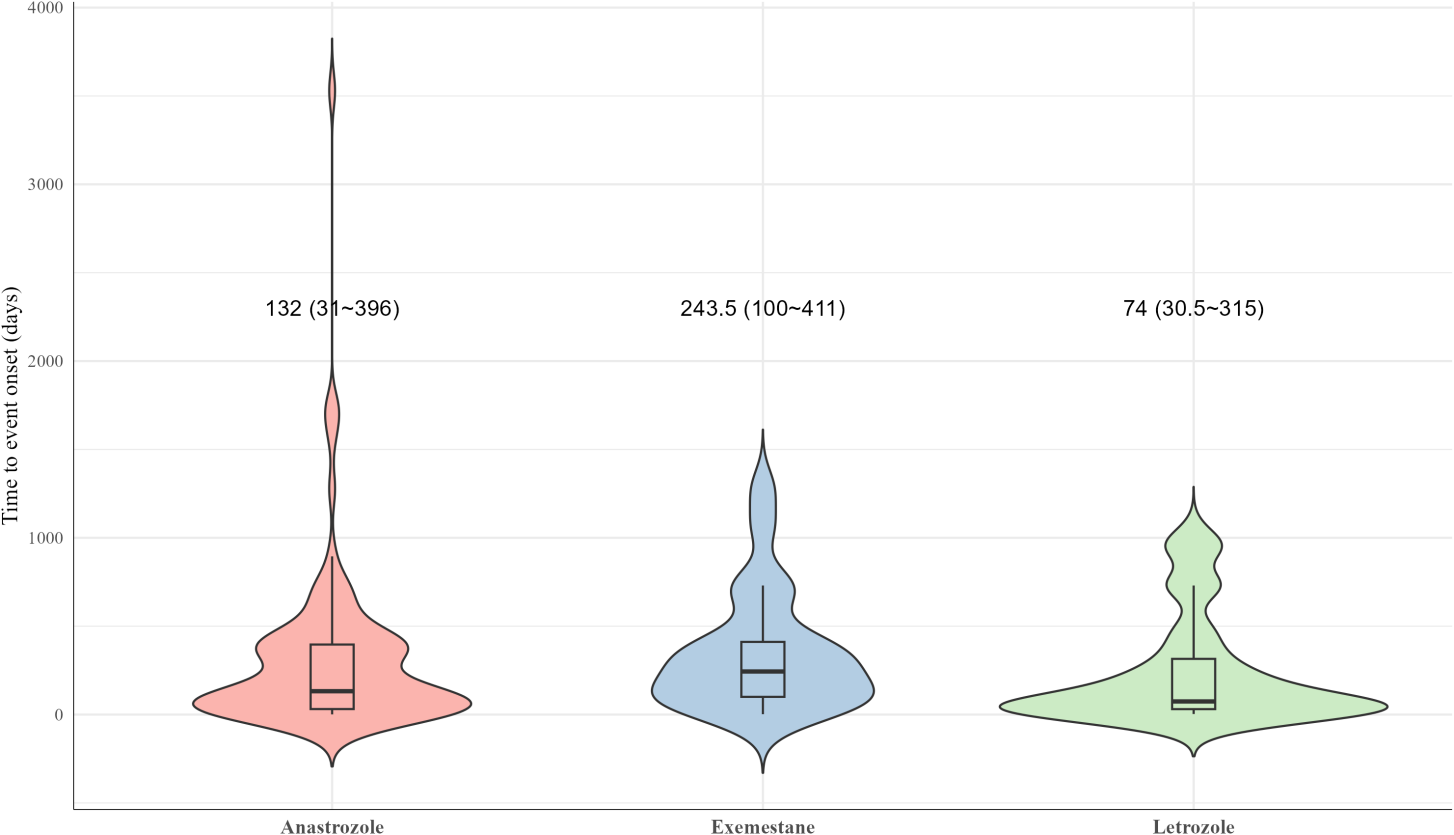
Time to onset of tendon disorders. The X-axis represents different drugs, including Anastrozole, Exemestane, and Letrozole. The Y-axis represents the time to event onset, measured in days. The violin plots depict the distribution of the data, illustrating the number of patients corresponding to each onset time.

The occurrence timing of third generation AIs related tendon disorders was gathered from the database, while records with unreported or unknown occurrence times were excluded. **Figure 4** depicts the onset time of tendon disorders for various third generation AIs. Letrozole demonstrated an earlier median onset time, at 74 days (IQR: 30.5–315 days). Conversely, the longest was 243.5 days (IQR: 100–411 days) for exemestane. Anastrozole presented the widest range, at a median time of 132 days (IQR: 31–396 days).

### 3.5 Sensitivity analysis

To mitigate the influence of potential confounders, such as medication combinations, we presented the tendon disorders risks of the top 10 concomitant drugs in **Supplementary Table 2**. The principal combined drugs involving these three medications encompassed ribociclib, palbociclib, trastuzumab, fulvestrant, denosumab, zometa acid, calcium, everolimus, aspirin and abemaciclib. Subsequently, we performed stratification analysis by combined use (combination therapy and without combination therapy), the concomitant medication was defined as the combined use of at least one or more of the 10 drugs listed above. As shown in **Table 4**, stratification analysis revealed that both combination therapy and non-combination therapy groups consistently exhibited positive disproportionation signals for tendon disorders across all three aromatase inhibitors. For anastrozole, the combination therapy group showed an ROR of 8.06 (95% CI: 5.00–12.99), PRR of 8.01 (χ² = 104.33), EBGM05 of 5.37, and IC025 of 1.33, while the non-combination therapy group yielded an ROR of 7.66 (95% CI: 6.91–8.51), PRR of 7.62 (χ² = 2056.59), EBGM05 of 6.94, and IC025 of 1.25. For letrozole, the combination therapy group demonstrated an ROR of 6.23 (95% CI: 4.63–8.38), PRR of 6.20 (χ² = 191.99), EBGM05 of 4.84, and IC025 of 0.97, whereas the non-combination therapy group showed an ROR of 3.45 (95% CI: 3.03–3.93), PRR of 3.44 (χ² = 392.29), EBGM05 of 3.08, and IC025 of 0.11. For exemestane, the combination therapy group had an ROR of 4.85 (95% CI: 2.18–10.82), PRR of 4.83 (χ² = 18.26), EBGM05 of 2.47, and IC025 of 0.61, while the non-combination therapy group showed an ROR of 4.42 (95% CI: 3.59–5.44), PRR of 4.41 (χ² = 234.13), EBGM05 of 3.70, and IC025 of 0.47. These findings indicate that positive signals for tendon disorders persist even after excluding the effect of concomitant medications, confirming that the observed association is not merely driven by drug interactions or polypharmacy. Therefore, the concurrent use of other pharmaceuticals did not bias the results, although it may have amplified the apparent strength of the link in cases involving concurrent medications. Stratification strategies were employed to enhance the robustness of the results.

**Table 4:** Sensitivity analysis of tendon disorders of third generation AIs.

Therapy regimens	Cases of interest	ROR (95% Cl)	PRR (χ2)	EBGM (EBGM05)	IC (IC025)
Anastrozole Combination Therapy	17	8.06( 5 - 12.99 )	8.01 ( 104.33 )	8.01 ( 5.37 )	3( 1.33 )
Anastrozole without Combination Therapy	360	7.66( 6.91 - 8.51 )	7.62( 2056.59 )	7.57 ( 6.94 )	2.92( 1.25 )
Letrozole Combination Therapy	44	6.23( 4.63 - 8.38 )	6.2 ( 191.99 )	6.2( 4.84 )	2.63 ( 0.97 )
Letrozole without Combination Therapy	227	3.45( 3.03 - 3.93 )	3.44( 392.29 )	3.43( 3.08 )	1.78 ( 0.11 )
Exemestane Combination Therapy	6	4.85( 2.18 -10.82 )	4.83( 18.26 )	4.83( 2.47 )	2.27 ( 0.61 )
Exemestane without Combination Therapy	89	4.42 ( 3.59 - 5.44 )	4.41( 234.13 )	4.4( 3.7 )	2.14 ( 0.47 )

AIs, aromatase inhibitors.

## 4 Discussion

Hormonal manipulation represents the major therapeutic approach for women with BC expressing estrogen and progresteron (27). AIs have radically changed the prognosis of hormone receptor positive BC in post-menopausal women, and are a mainstay of the adjuvant therapy for BC after surgery in place of, or following, Tamoxifen (28). Because of their excellent tolerability and great efficacy, AIs have been regarded as first-line therapy for patients with hormone-dependent BC (29). However, the underlying tendon disorders of AIs is frequently overlooked. By conducting a retrospective pharmacovigilance analysis based on the FAERS data from the previous few years, our study offers a comprehensive insight into the potential tendon disorders associated with different third generation AIs. Following data cleansing and deduplication, a total of 664 reports regarding the tendon disorders of third generation AIs were identified. This represents the first large-scale postmarketing data analysis examining the association between third generation AIs and tendon disorders.

Of the known reported cases, the incidence is significantly higher in females than in males, which correlates with the more common use of third-generation aromatase inhibitors in female BC patients (30). Notably, three drugs were implicated in the occurrence of these tendon disorders, and the bulk of cases that were reported came from European and American nations. This might be as a result of the earlier third-generation AIs available to these countries and the priority placed on reporting adverse drug events. Furthermore, based on the number of ADEs linked to tendon disorders that have been reported over time, it can be observed that letrozole has been linked to a significant increase in tendon disorders over the past five years. This suggests that while we should be aware of the possibility that letrozole may cause tendon disorders, we also shouldn’t overlook the possibility that similar medications like exemestane and anastrozole may cause tendon disorders in ADEs. This observation aligned with the department of Health’s recent cautions in Canada about the potential risk of tendon disease with third generation AIs. It also underscored the reliability of the FAERS data and the viability of the study methodology.

This study, based on real-world data mining, is the first to consolidate signals related to tendon disorders concerning third generation AIs. Although the number of reports for exemestane was lower than for anastrozole and letrozole, significant ADE signal intensity was still observed for tendon disorders. The study underscored a positive correlation between these three drugs and the occurrence of tendon disorders. Although well tolerated, AIs are not completely free of adverse events, in this regard, a number of reports involved the musculoskeletal system, AI-associated arthralgia (AIA) syndrome and autoimmune rheumatic diseases (31). Musculoskeletal symptoms induced by AIs include arthralgia, joint stiffness, bone pain, bone fracture, and an increased risk of osteoporosis (17). These symptoms can be severe in a third of the patients. Carpal tunnel syndrome, tenosynovitis (18), and tendinopathy are less frequently recorded (19). According to the FDA-approved product labels for anastrozole (NDA 020541; Available at: https://www.accessdata.fda.gov/drugsatfda_docs/label/2024/020541s036lbl.pdf), letrozole (NDA 020726; Available at: https://www.accessdata.fda.gov/drugsatfda_docs/label/2024/020726s043lbl.pdf), and exemestane (NDA 020753; Available at: https://www.accessdata.fda.gov/drugsatfda_docs/label/2024/020753s025lbl.pdf), as well as the published results of major phase III clinical trials (ATAC trial and BIG 1–98 trial), none of these sources have included specific warnings regarding tendon disorders or tendinopathy. Consequently, AI-associated tendinopathy and muscle tendon rupture remain exceedingly rare in the literature. The primary explanation for the paucity of data on adverse reactions to tendon disorders are, firstly, the low incidence of the adverse reaction itself and the lack of clinical attention; secondly, the diagnosis of tendon disorders requires a more specialized assessment without the visual manifestation of symptoms, which may not be easier for patients and physicians to recognize and report; and finally, the relatively long median onset time of tendon disorders leads to neglect. Up to date, only Martens HA reported a case of anastrozole-associated severe tendonitis and Nikolaos M et al. reported three cases of tendonitis or tendon rupture in postmenopausal hormone receptor-positive BC patients treated with letrozole in the medical literature, and there are no recorded cases of AI-associated muscle tendon rapture (32, 33).

However, we raised concerns regarding the potential risk of tendon disorders associated with third generation AIs, as indicated in PT cluster analysis. Our analysis revealed varying types of tendon toxicity risk associated with three drugs at the PT level, along with novel AE signals not covered by the drug labels observed in three drug. All the drugs had a significant correlation with tenosynovitis stenosans, trigger finger, tendonitis and tenosynovitis, particularly the first two. Additionally, new signal of tendinous contracture was discover only in anastrozole, exemestane also presented a certain degree of tendon toxicity, with new signals of tendon discomfort and tendon disorder identified at the PT level. Ovarian suppression therapy (e.g., GnRH agonists) is typically used in premenopausal patients, while third-generation AIs are indicated for postmenopausal breast cancer. Given that the most patients in each drug group were aged ≥45 years, consistent with a postmenopausal status, ovarian suppression therapy is not a standard concomitant treatment (**Table 2**). Moreover, none of the top 10 concomitant medications identified in our dataset (**Supplementary Table 2**) included ovarian suppression agents. Therefore, potential confounding from this therapy is unlikely to affect our findings.

Mechanistically, estrogen is mostly produced in the ovaries before menopause. Following menopause, the P450 cytochrome enzyme aromatase converts testosterone to estrogen, primarily producing estrogen in peripheral tissues such as the skin, fat, muscle, and benign and malignant breast tissue (34). By blocking or deactivating aromatase, the enzyme that converts androgens to estrogens peripherally, AIs lower plasma estrogen levels. A competitive inhibitor of aromatase is provided by the non-steroidal AIs, letrozole and anastrozole. Aromatase is irreversibly inhibited by the steroidal AI exemestane. However, both non-steroidal AIs and steroidal AI can reduce tissue and plasma levels of estrone, the primary estrogen in postmenopausal women. It is yet unknown what causes AIs-associated tendon disorders. There are two hypotheses: the first is that, the retinaculum, the bands that surround the tendons, and the intermediate layer of the annular ligaments, which make up the flexor pulley tendons, have been shown to contain estrogen receptors. Tenosynovitis may arise as a result of changes to these tendons brought on by the aromatase inhibitors’ blockage of these receptors. There is a link between the consumption of AIs and tendinopathy, or rupture of the muscle tendon, even though there is no clear pathophysiological mechanism (20). The second is that, rheumatologic diseases, inherited disorders, and endocrine and metabolic disorders can all be high-risk factors for the development of tendinopathy. Inherited disorders can result in deficient or abnormal collagen or abnormal fibril structure, while endocrine and metabolic disorders can alter collagen metabolism or deposits between fibrils (35). Despite the differences in pharmacological classification, non-steroidal reversible inhibitors (anastrozole, letrozole) versus a steroidal irreversible inhibitor (exemestane), all three third-generation AIs effectively reduce peripheral and systemic estrogen levels. Estrogen receptors have been identified in tendon-associated structures such as the retinaculum and annular ligaments (20). Therefore, estrogen deprivation induced by any of these agents may contribute to tenosynovitis or tendinopathy, supporting a class effect. The variation in the number of reported cases across drugs likely reflects differences in clinical utilization and spontaneous reporting patterns rather than a fundamental difference in tendon toxicity potential. In contrast, tamoxifen, as a selective estrogen receptor modulator, exhibits tissue-specific estrogenic activity in the musculoskeletal system, which may contribute to its relatively lower risk of tendinopathy compared with AIs (19). This distinction underscores the importance of recognizing AI-specific tendon toxicity in clinical practice.

When weighed against other types of adverse events, such as liver damage, cardiotoxicity, interstitial lung disease, and thromboembolism, tendon disorders are frequently disregarded and may not have a major impact on treatment choices. However, severe tendinopathy or rupture, and the thromboembolism or surgical risks it entails, can be fatal, with serious and possibly permanent consequences. Because tendon diseases are uncommon, it is extremely important from a clinical standpoint to quickly distinguish between different kinds of these reactions. It is essential to stop using the drugs in question as soon as possible and to take the right management steps. Our study revealed that although tendon disorders ADE are all long-lasting, their chronic nature means that they shouldn’t be clinically disregarded. The median time of onset of anastrozole, letrozole, and exemestane leading to associated tendon disorders unearthed in this study provides new clues to the possible time of onset of tendon disorders due to the clinical application of third-generation AIs. In this study, anastrozole and letrozole show very different profiles of association with tendon disorders and time of onset of tendon disorders. Differences in the drug’s metabolic profile could be the cause: Anastrozole has a half-life of 50 hours and achieves its maximal plasma concentration in 2 hours. It is metabolized in the liver and eliminated in the urine as either catabolites (60%) or the unaltered drug (10%) after reaching steady-state concentrations after 7–10 days (36). Although letrozole has a 42-hour half-life, steady-state concentrations can be reached after 2–6 weeks of treatment (37). Because of its lengthy half-life and delayed clearance in the body, anastrozole may cause long-lasting side effects, and lead to differences in their level of risk for tendon disorders and time of onset of tendon disorders. This study provides physicians with a general window of time during which the incidence of possible tendon disorders should be emphasized. Additionally, it was also a crucial instrument for determining and evaluating possible principal suspected drug.

Drug-induced tendon disorders has most commonly been associated with fluoroquinolones, statins and glucocorticoids (38). Moreover, amlodipine, anabolic steroids, antiretrovirals, isotretinoin, renin-angiotensin II receptor antagonists, rituximab, and sitagliptin are among the other systemic medications that have been linked to tendinopathy (20, 39). Albeit less frequent, other oral antibiotics, including cephalosporins, azithromycin, and sulfonamides, have also been associated with toxic tendinopathy. Notably, AIs have often been disregarded as possible culprits for causing tendon disorders. This oversight may arise from the scarcity of relevant reports, particularly in scenarios involving multiple suspected drugs. In this study, the main confounding factors were the co-administered drugs, to reduce these possible biases and improve the reliability of our results, subsequently, we analyzed the top 10 concomitant medications were stratified by combination therapy and without combination therapy. Excitingly, our study detected the AIs-related tendon disorders and found that the three drugs all showed positive significant disproportionation signals. Hence, the association between the target drugs and tendon disorders remained when stratified by combined use. All three drugs showed the same results: positive signals value were higher in the combination therapy group than in the without combination therapy group. Additionally, it showed that there appears to be a stronger correlation between tendon diseases and third-generation AIs when concurrent drugs are taken. It’s worth noting that postmenopausal women may combine the use statins, renin-angiotensin II receptor antagonists and other “high-risk drugs”, which raise the possibility of tendon toxicity and necessitate medical attention. Moreover, risk factors associated with aromatase inhibitor tenosynovitis include prior chemotherapy(particularly with taxanes). In every case of a female patient with hormone receptor-positive BC under treatment with AIs and arthralgia, further testing should be performed in order to exclude the presence of tendinopathy or muscle tendon rupture. Thus, for patients receiving AIs therapy, any potential additive tendon toxicity requires further investigation to ascertain the true risk.

From a clinical perspective, the practical value of this pharmacovigilance analysis lies in its ability to guide early recognition and risk stratification of AI-associated tendon disorders. Although joint symptoms are commonly monitored during AI therapy, tendon-specific complaints such as trigger finger or stenosing tenosynovitis are often overlooked until functional impairment occurs. Our finding that anastrozole demonstrates the strongest disproportionality signal suggests that patients on this agent may warrant closer surveillance for tendinopathy. Furthermore, the distinct time-to-onset profiles, earliest with letrozole (median 74 days) and latest with exemestane (median 243.5 days), offer a practical timeline for clinicians to educate patients on when to remain vigilant. In particular, the first three to eight months of AI treatment appears to be a critical window for monitoring tendon symptoms. These insights support the integration of tendon-specific assessments into routine endocrine therapy follow-up protocols, especially for postmenopausal women with hormone receptor-positive breast cancer.

This study delved into the correlation between third generation AIs in relation to tendon disorders from two distinct perspectives. Firstly, grounded in the premise that the third generation AIs were the primary suspected drugs, the positive signal for drug-associated tendon disorders suggested a connection between third-generation aromatase inhibitors and reported adverse events of tendon disorders. This elucidated whether these three drugs bore an association with tendon disorders. Furthermore, this study undertook an initial exploration of the combined usage of third generation AIs as potential triggers for tendon disorders. This investigation served as a prompt for medical practitioners, signaling the potential for tendon disorders to arise when these three drugs are administered in tandem with “high-risk drugs” medications. This awareness changed the inherent influence of clinicians, suggested not to ignore the fact that third generation AIs could lead to tendon disorders, and enriched the catalog of primary suspected drugs implicated in tendon disorders within the clinical setting.

However, this retrospective study has certain inherent limitations that should be acknowledged. First, the disproportionality analysis used in pharmacovigilance studies cannot establish causality but only generate hypotheses regarding potential associations (40, 41). Second, the FAERS database, as a spontaneous reporting system, is subject to multiple biases, including underreporting (only a fraction of actual adverse events are ever reported), reporting bias (the likelihood of reporting may be influenced by media attention or regulatory actions, such as the Weber effect), and notoriety bias (increased reporting following published case reports or safety warnings) (42, 43). Third, the database lacks a denominator, preventing the calculation of true incidence rates, and does not provide detailed information on diagnostic methods, clinical signs and symptoms, or objective confirmation of tendon disorders (44). Fourth, confounding by indication and channeling bias may be present, as patients receiving different aromatase inhibitors may have distinct clinical characteristics or risk profiles that influence both drug selection and the occurrence of tendon disorders (45). Fifth, the absence of accurate disease staging and treatment history (e.g., prior chemotherapy with taxanes) limited our ability to perform stratified analyses on these important risk factors. Finally, missing or inaccurate data (e.g., onset time, concomitant medications, body mass index) are common in spontaneous reporting systems and may introduce information bias (46). Despite these limitations, our study provides a systematic and methodologically rigorous assessment of tendon toxicity signals associated with third-generation aromatase inhibitors, offering valuable real-world evidence to guide clinical monitoring and future hypothesis-driven research.

## 5 Conclusion

In conclusion, our study identified positive disproportionality signals for tendon disorders associated with all three third-generation AIs, supporting a class effect despite variations in the absolute number of reported cases. Anastrozole showed the strongest signal, followed by exemestane and letrozole. At the preferred term level, trigger finger and tenosynovitis stenosans were the most significant signals across all three drugs. The median time to onset varied considerably, with letrozole presenting the earliest onset and exemestane the latest. Sensitivity analysis confirmed that the association remained significant even after excluding the effect of concomitant medications. Despite some limitations inherent to the FAERS database, our findings support ongoing monitoring of tendon disorders during AIs therapy. Further evidence is necessary to confirm these associations; nevertheless, our findings support the clinical recommendation that tendon-specific symptoms, particularly trigger finger and tenosynovitis stenosans, should be actively monitored during third-generation AI therapy. Clinicians are advised to consider earlier onset with letrozole and stronger signal strength with anastrozole when selecting and monitoring these agents in postmenopausal breast cancer patients.

## Data Availability

The original contributions presented in the study are included in the article/**Supplementary Material**. Further inquiries can be directed to the corresponding author.

## Supplementary material

Supplementary Table 1 Four grid table of the proportional imbalance methods. a, number of reports containing both the target drug and target adverse drug reaction; b, number of reports containing other adverse drug reaction of the target drug; c, number of reports containing the target adverse drug reaction of other drugs; d, number of reports containing other drugs and other adverse drug reactions; N: the number of all reports.

Supplementary Table 2 Concomitant drugs with third generation AIs. AIs, aromatase inhibitors.
