# Supplementary material for: Unveiling the tendon toxicity spectrum of anastrozole, letrozole, and exemestane: a real-world pharmacovigilance study
Source: Front Oncol. 2026 Jul 7;16:1825180. doi: 10.3389/fonc.2026.1825180 (PMC13385238; doi:10.3389/fonc.2026.1825180)
Supplement: Supplementary Table 1 — Four grid table of the proportional imbalance methods. a, number of reports containing both the target drug and target adverse drug reaction; b, number of reports containing other adverse drug reaction of the target drug; c, number of reports containing the target adverse drug reaction of other drugs; d, number of reports containing other drugs and other adverse drug reactions; N: the number of all reports. [file DataSheet1.docx]

**Supplementary Table S1. Four grid table of the proportional imbalance methods.**

| **Drug class** | **Number of reports of the target adverse events** | **Number of reports of the other adverse events** | **Total** |
| --- | --- | --- | --- |
| Target drug | a | b | a+b |
| Other drugs | c | d | c+d |
| Total | a+c | b+d | N=a+b+c+d |

Note: a, number of reports containing both the target drug and target adverse drug reaction; b, number of reports containing other adverse drug reaction of the target drug; c, number of reports containing the target adverse drug reaction of other drugs; d, number of reports containing other drugs and other adverse drug reactions; N: the number of all reports.

**Supplementary Table S2. Concomitant drugs with third generation AIs.**

| **Concomitant drugs** | **N (%)116700** |
| --- | --- |
| Ribociclib | 10991(9.42%) |
| Palbociclib | 6452(5.53%) |
| Trastuzumab | 2658(2.28%) |
| Fulvestrant | 2125(1.82%) |
| Denosumab | 1853(1.59%) |
| Zometa acid | 1731(1.48%) |
| Calcium | 1451(1.24%) |
| Everolimus | 1322(1.13%) |
| Aspirin | 1229(1.05%) |
| Abemaciclib | 832(0.71%) |
